# A Rare Case of Right Testicular Leiomyosarcoma With Hepatic and Pulmonary Metastases

**DOI:** 10.7759/cureus.96099

**Published:** 2025-11-04

**Authors:** Kavi Chakravarthi M G, A Ravichandran, Yatish M Ellini, Kavuru Naga Siri, Bhargav Vuppumalla

**Affiliations:** 1 Medical Oncology, Sri Ramachandra Institute of Higher Education and Research, Chennai, IND; 2 Internal Medicine, Venkateshwara Hospitals, Chennai, IND

**Keywords:** first and second line chemotherapy, hepatic metastasis, oncology case report, palliative chemotherapy, pulmonary metastasis, rare case report, synchronous cancers, testicular leiomyosarcoma

## Abstract

Testicular leiomyosarcoma is an exceptionally rare malignant mesenchymal tumour with limited documentation in the literature. We report the case of a 50-year-old male presenting with progressive scrotal swelling and pain, who was diagnosed with right testicular leiomyosarcoma with synchronous hepatic and pulmonary metastases. The patient underwent right high orchiectomy, followed by systemic chemotherapy. Disease progression occurred after anthracycline-ifosfamide, requiring second-line docetaxel. This case highlights the aggressive clinical course and therapeutic challenges of testicular leiomyosarcoma, underscoring the need for more cumulative evidence to guide management.

## Introduction

Primary tumours of mesenchymal origin in the testis are exceedingly rare, accounting for <2% of testicular neoplasms [1]. Leiomyosarcoma, a malignant neoplasm of smooth muscle differentiation, typically arises in the spermatic cord, epididymis, or tunical structures [2]. Due to its rarity, optimal management strategies are undefined, and treatment decisions are often extrapolated from guidelines for soft tissue sarcoma [3,4]. Radical inguinal orchiectomy is the treatment of choice, which is followed by monitoring for recurrence. Here, we present a case of metastatic testicular leiomyosarcoma in a middle-aged male who underwent right high orchiectomy for debulking of the disease. Due to the rarity of tumour, optimal therapeutic management is not well defined; by adding to the sparse literature, our goal is to assist in the better definition of the right treatment strategy for such cases.

## Case presentation

A 50-year-old male, with no prior comorbidities, presented with progressive right scrotal swelling for 18 months and pain for one month. He denied constitutional symptoms. Family history was unremarkable. On physical examination, a firm right testicular mass was noted. Systemic examination was within normal limits.

Imaging

Ultrasound Imaging

**Figure 1:**
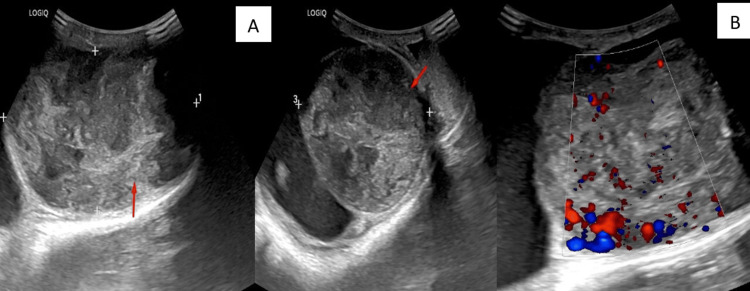
Two-dimensional B-mode ultrasound image and colour Doppler image Bulky testis entirely replaced by heteroechoic mass lesion (red arrows in A) with increased vascularity (B).

Bulky testis entirely replaced by heteroechoic mass lesion (red arrows in Figure 1A) with increased vascularity (Figure 1B).

CT Imaging

**Figure 2:**
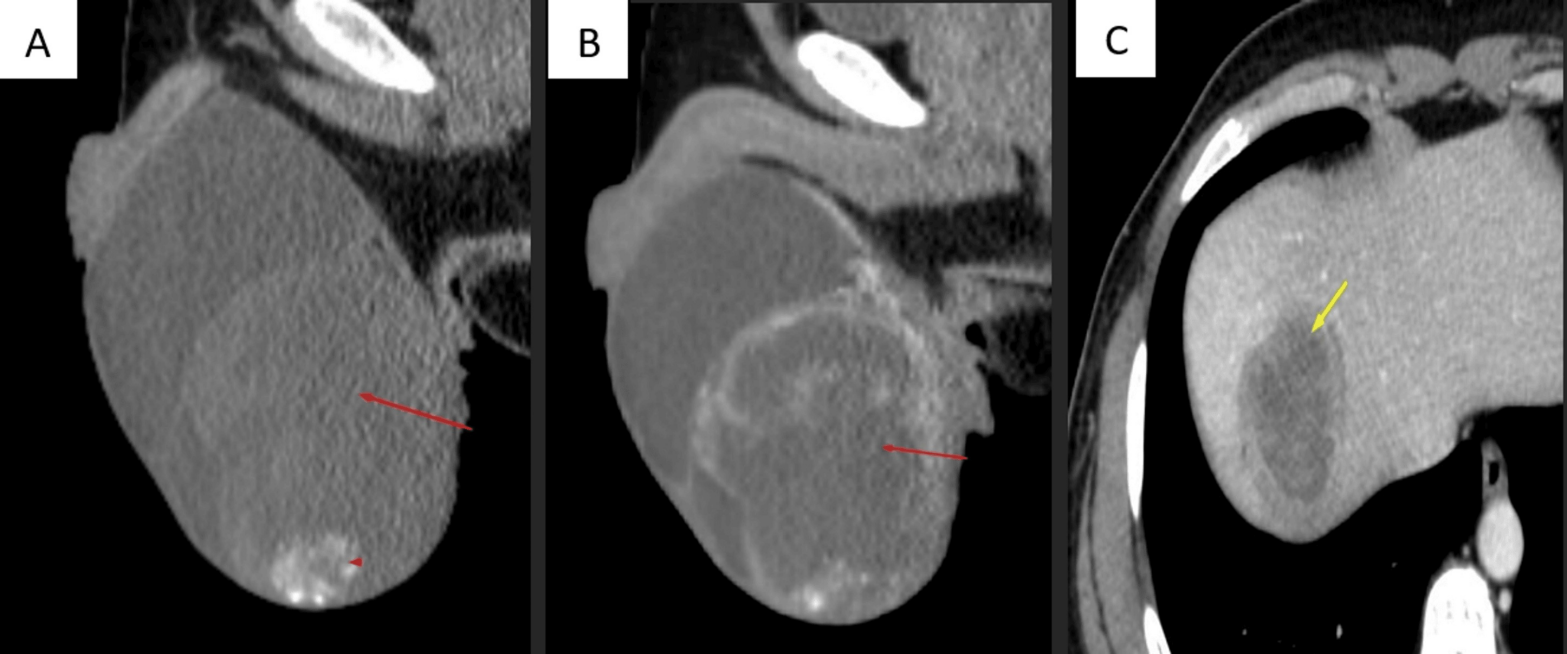
Unenhanced CT at sagittal plane, contrast-enhanced CT in venous phase at sagittal plane, and contrast-enhanced CT in venous phase at axial plane Unenhanced CT at sagittal plane showing enlarged testis with heterodense mass (red arrow) with calcific specks (arrowhead) in inferior aspect (A). Contrast-enhanced CT in venous phase at sagittal plane showing heterogenous contrast enhancement (red arrow) (B). Contrast-enhanced CT in venous phase at axial plane showing hepatic metastasis (yellow arrow) in right lobe of liver (C).

Unenhanced CT at sagittal plane showing enlarged testis with heterodense mass (red arrows) with calcific specks (arrowhead) in inferior aspect (Figure 2A). Contrast-enhanced CT in venous phase at sagittal plane showing heterogenous contrast enhancement (Figure 2B). Contrast-enhanced CT in venous phase at axial plane showing hepatic metastasis (yellow arrow) in right lobe of liver (Figure 2C).

PET-CT Imaging

**Figure 3:**
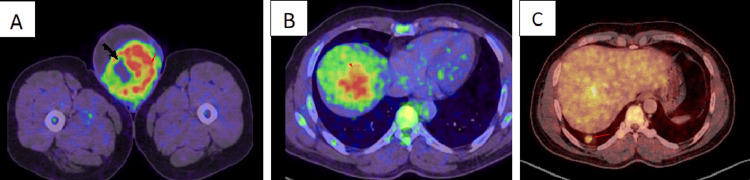
Axial post-processed fused PET-CT shows avid FDG uptake (A) Axial post-processed fused PET-CT shows avid FDG uptake within the solid cystic lesion of the left hemiscrotum (short arrow), (B) FDG-avid delayed-enhancing lesion in segment 7 of the liver (arrowheads), and (C) the largest nodule with FDG uptake in the right posterobasal segment (long arrow). PET/CT: positron emission tomography/computed tomography, FDG: fluorodeoxyglucose.

Baseline 18F-fluorodeoxyglucose positron emission tomography/computed tomography (18F-FDG PET/CT) (January 2025) revealed a 10.2 × 8.7 × 14.7 cm FDG-avid solid-cystic lesion in the right hemiscrotum (maximum standardized uptake value (SUVmax) 9.1) (Figure 3A). Multiple FDG-avid liver lesions revealed a largest 4.3 × 5.1 cm with FDG uptake (SUVmax 10.7) (Figure 3B). Pulmonary nodules revealed a largest 1.6 × 1.0 cm nodule with mild FDG uptake (SUVmax 3.9) (Figure 3C). No significant lymphadenopathy was noted.

Surgery and pathology

**Figure 4:**
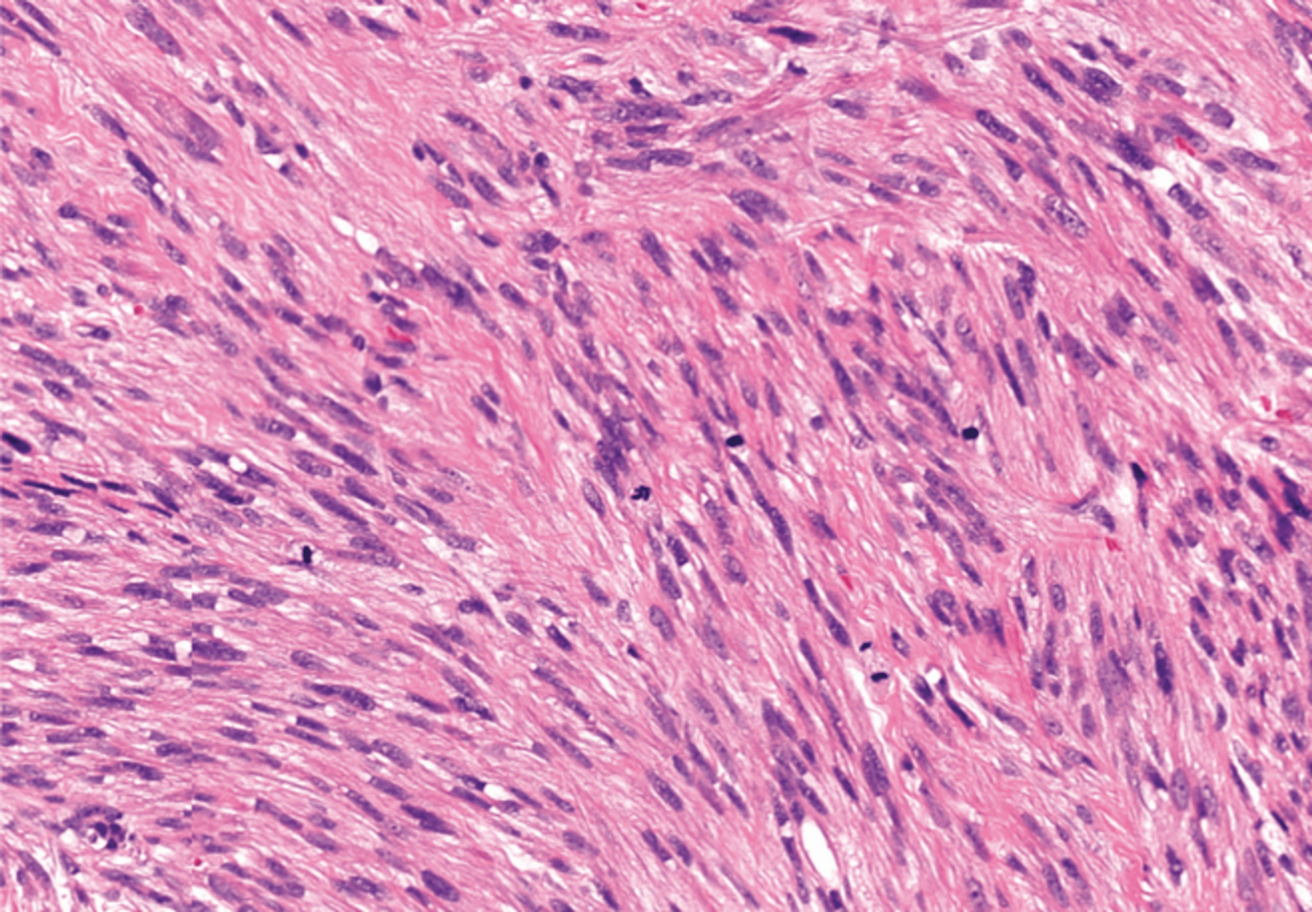
Spindle cell neoplasm composed of elongated cells with eosinophilic cytoplasm, pleomorphic nuclei, and mitoses

The patient underwent right high orchiectomy (January 1, 2025). Histopathology revealed a spindle cell neoplasm composed of elongated cells with eosinophilic cytoplasm, pleomorphic nuclei, and mitoses (Figure 4). Immunohistochemistry was positive for smooth muscle actin (SMA) and desmin, negative for germ cell markers (SALL4, PLAP), consistent with leiomyosarcoma.

Systemic therapy

The patient received six cycles of anthracycline-ifosfamide (AI regimen) as first-line therapy (palliative intent). Restaging showed progressive hepatic and pulmonary metastases. He was subsequently initiated on docetaxel monotherapy as second-line treatment. At the latest follow-up (September 2025), the patient maintained stable performance status on docetaxel.

## Discussion

Primary testicular leiomyosarcoma is rare, with fewer than 50 cases described in the literature [1,2]. It is thought to arise from smooth muscle elements of the spermatic cord, epididymis, or tunica. The differential diagnosis includes paratesticular sarcoma, rhabdomyosarcoma, and spindle cell sarcoma. Immunohistochemistry (IHC) is essential for diagnosis, confirming smooth muscle origin [3].

Localized disease is best treated with radical orchiectomy, which provides excellent local control [2]. However, advanced and metastatic disease requires systemic therapy, with evidence largely extrapolated from soft tissue sarcoma trials. Anthracycline ± ifosfamide remains the standard first-line chemotherapy, with modest response rates (20%-30%) and median overall survival of 12-18 months in metastatic soft tissue sarcoma [4]. Alternative regimens in later lines include gemcitabine-docetaxel, taxane monotherapy, and targeted therapies such as pazopanib [5].

Previous studies recommend extension assessment to be based on thoraco-abdominal-pelvic CT, to look for suspicious lymph nodes or metastasis, especially in lungs [6]. Adjuvant chemotherapy is only advised for advanced stage tumours only, the rest may be followed up by semi-annual pelvic CT scan [7,8]. Our patient had underwent a PET-CT scan initially, as he had a massive right scrotal swelling, with clinical manifestations of metastasis (shortness of breath, weight loss, and occasional night sweats).

Our patient presented with synchronous liver and lung metastases, which is exceedingly rare. The disease progressed on anthracycline-ifosfamide, necessitating second-line docetaxel. His course reflects the aggressive biology and limited efficacy of current systemic therapies.

## Conclusions

Testicular leiomyosarcoma is a rare, aggressive malignancy with poor outcomes in the metastatic setting. Reporting such cases is vital to improve understanding of its natural history and treatment responses. Individualized therapy, guided by soft tissue sarcoma protocols, remains the cornerstone of management until disease-specific guidelines can be established.

Few primary low-grade intratesticular tumours are mentioned in the literature, which suggests semi-annual pelvic CT to assess follow-up. We recommend the same, with a very low tolerance to undergo an interval pelvic CT any time a suspicion arises. If the patient is symptomatic, we suggest doing a high-resolution computed tomography (HRCT) chest.

Due to the aggressive nature of the malignancy, even if histopathology comes back as a low-grade malignancy, we suggest aggressively following up the patient, as a means to avoid upfront secondary presentation with multiple metastases. Our case report highlights the aggressive malignancy with much poorer outcomes in the metastatic setting.

## References

[1] Daugaard G (2003). Sarcomas of the testis and paratesticular region. APMIS.

[2] Mearini L, Colella R, Zucchi A, Porena M (2007). Leiomyosarcoma of the spermatic cord: review of the literature. Urol Int.

[3] Andrew L, Folpe G, Nielsen P (2014). Enzinger and Weiss's Soft Tissue Tumors. 6th Edition. https://shop.elsevier.com/books/enzinger-and-weisss-soft-tissue-tumors/folpe/978-0-443-24875-7.

[4] Judson I, Verweij J, Gelderblom H (2014). Doxorubicin alone versus intensified doxorubicin plus ifosfamide for first-line treatment of advanced or metastatic soft-tissue sarcoma: a randomised controlled phase 3 trial. Lancet Oncol.

[5] (2025). NCCN Clinical Practice Guidelines in Oncology (NCCN Guidelines®): Soft tissue sarcoma. https://www.nccn.org/guidelines/guidelines-detail?category=1&id=1464.

[6] Abdallah H, Dergamoun H, Hachem F, Boughaleb A, Al Sayegh H, Nouini Y (2021). Testicular leiomyosarcoma: a case report and literature review. Int J Surg Case Rep.

[7] Siraj F, Sharma S, Rai CB, Vasudeva P (2018). Primary high grade testicular leiomyosarcoma: a rare malignancy in a young male. Turk J Urol.

[8] Abdullazade S, Kara O, Akdoğan B, Baydar DE (2013). Primary low grade intratesticular leiomyosarcoma: case report and review of the literature. Turk Patoloji Derg.

